# Somatic-vegetative Symptoms Evolution in Postmenopausal Women Treated with Phytoestrogens and Hormone Replacement Therapy

**Published:** 2017-11

**Authors:** Delia Mirela ŢIŢ, Annamaria PALLAG, Ciprian IOVAN, Gheorghe FURĂU, Cristian FURĂU, Simona BUNGĂU

**Affiliations:** 1. Dept. of Pharmacy, Faculty of Medicine and Pharmacy, University of Oradea, Oradea, Romania; 2. Dept. of Preclinical Disciplines, Faculty of Medicine and Pharmacy, University of Oradea, Oradea, Romania; 3. Dept. of General Medicine, Faculty of Medicine, Western University “Vasile Goldiş” of Arad, Arad, Romania; 4. Dept. of Life Sciences, Faculty of Medicine, Western University “Vasile Goldiş” of Arad, Arad, Romania

**Keywords:** Phytoestrogens, HRT, Evaluation, Post menopause

## Abstract

**Background:**

The purpose of this study was to compare the effects of the phytoestrogens in relieving and/or suppressing the specific somatic-vegetative symptoms of menopause with those of the hormone therapy, administered in small doses.

**Methods:**

The study was conducted in the County Clinical Emergency Hospital Oradea - Obstetric-Gynecological Ambulatory, and in private obstetrics-gynecology cabinets from Bihor County (NW Romania), from November 2011 to January 2014. Overall, 325 patients clinically diagnosed with specific postmenopausal symptomatology and not previously treated with phytoestrogens or hormone replacement therapy (HRT), were divided into 3 groups. Symptom assessment was performed with a standardized questionnaire named Menopause Rating Scale (MRS) in three phases: beginning of the treatment, after six months, and after one year. The administered doses for three different groups were as follows: 1 mg estradiol and 0.5 mg norethisterone acetate (NETA) p.o. daily (i.e. for the HRT group); 40 mg of isoflavones p.o. daily - i.e. 2 capsules of 40% standardized extract containing 20 mg of pure isoflavones: Genistein, Daidzein, and Glycitein (i.e. for the group with phytoestrogens); and no trreatment for the control group.

**Results:**

The evolution of the somatic-vegetative symptoms was better in both groups treated either with phytoestrogens or HRT (*P*<0.001) as opposed to the higher percentage of patients with stationary symptoms in the control group (i.e. 37.74% for control group, 16.13% for the group treated with phytoestrogens, respectively 18.95% for the group treated with HRT).

**Conclusion:**

Twelve months treatment study is a favorable evidence for the use of soy derived phytoestrogens in the treatment of somatic-vegetative symptoms at postmenopausal women.

## Introduction

Hormonal changes in post menopause are associated with numerous physical and psychological symptoms with an increased risk for several chronic diseases, including osteoporosis, cardiovascular disease and loss of cognitive function (1).

Typical symptoms of menopause are hot flashes, vaginal dryness (2) and sleep disorders (3). The likelihood of having other symptoms is greater when the woman has vasomotor symptoms (2). Approximately, 90% of women in the premenopausal stage and 60%–80% of postmenopausal stage are affected by this type of symptoms (4). The general term of Menopause is defined by three distinct stages: pre menopause, menopause and post menopause. Premenopause is a transition period that precedes menopause itself, characterized by the decreasing of the fertility till the fertility completely disappears. Menopause is diagnosed retrospectively after 12 months of amenorrhea and reflects an almost complete cessation of ovarian hormone secretion. Postmenopause is the last stage of the menopause cycle and lasts until the ovarian functional activity is completely stopped (i.e. 2–6 yr) (5).

For a long time, the estrogens alone or in combination with progestins, compounds with progesterone-like biological activity, such as NETA, represented the elective therapy for the relief of menopausal symptoms (6, 7). The results of two large studies on HRT, Heart and Estrogen/Progestin Replacement Study (HERS) and the Women’s Health Initiative (WHI) (8, 9) have changed the perception of the risks and/or benefits of this therapy, indicating an increased risk of venous thrombotic disease, breast cancer, stroke and coronary heart disease. Prescription and the use of HT are still controversial and require evaluation of the benefits of treatment against the potential risks (10, 11).

In this study, HRT was administered daily as a combined preparation with a low dose of hormones, a fixed combination of estrogen and progestin (0.5 mg estradiol and 1 mg NETA) recommended to women, in menopause for at least one year.

The present study intended to improve the treatment of some symptoms of the menopause by replacing the estroprogestative pharmaceutical products, with products containing phytoestrogens, estrogenic action phytochemicals, because they have fewer contraindications and side effects, high efficiency and cover a wide range of clinical manifestations caused by the installation of the menopause. The objective of this study was to compare in vivo the effects of low dose HRT and phytoestrogens in improving the somatic-vegetative symptoms in postmenopausal women.

## Materials and Methods

The study was conducted in the County Clinical Emergency Hospital Oradea - Obstetric-Gynecological Ambulatory, and in private obstetrics-gynecology cabinets from Bihor County (NW Romania), from November 2011 to January 2014. It was managed over a period of one year (12 months) for each patient, on three parallel groups, on 325 postmenopausal women clinically diagnosed with specific symptoms, not previously treated with HRT or phytoestrogens (5).

In accordance with the WMA Declaration of Ethical Helsinki-Medical Research Involving Human Principles for Subjects approved by the Ethics Committee of the Faculty of Medicine and Pharmacy, from the University of Oradea.

The distribution of patients was performed taking into account the patient’s history, assessment, and diagnosis, as well as the risks and benefits of the suggested treatment.

A sample of 95 patients was treated with HRT (1 mg estradiol and 0.5 mg NETA p.o. daily). A sample of 124 patients has treated with phytoestrogens 40 mg of isoflavones p.o. daily (2 capsules of 40% standardized extract containing 20 mg of pure isoflavones: Genistein, Daidzein, and Glycitein), and a sample of 106 patients belonged to the control group (i.e. with no treatment) (5, 12).

The symptoms evaluation was done with the help of a questionnaire, named Menopause Rating Scale (MRS), projected and standardized as a tool for self-assessment to quantify the changes in pre- and postmenopause hormone replacement therapy; the questionnaire consisted of 11 items, divided into three areas: somatic-vegetative, psychological and urogenital. We assessed the somatic-vegetative field, which contains 4 items: hot flashes, and sweating (episodes of sweating), heart problems (unusual awareness of the heartbeat, temporary heartbeat interruptions, rapid heartbeat, tightness), sleep disorders (difficulties in falling asleep and difficulties in sleeping all night, wake up earlier than usual) and unpleasant sensations in joints and muscles (joint pains, rheumatic symptoms).

For each item, the severity of symptoms is expressed from 0 to 4 points. The total field score was realized by summing the score of each item. The evaluation was done as follow (for both the area and each item): the score 0 – the absence of any symptom; the decrease of the previous score - improvement; and the increase of the previous score – worse (5, 12).

We considered as favorable evolution both the relieve symptoms as well the disappearing of the symptom.

All patients were initially evaluated, at 6 months and at 12 months from the starting of the treatment. Each patient included in the study signed an informed consent form (5, 12).

A statistical analysis was realized. For this purpose, the EPIINFO, version 6.0 (a program of the CDC - Center for Disease Control and Prevention in Atlanta, adapted to processing of medical statistics) was used (5, 12).

## Results

No significant differences were observed (*P*=0.712), between the groups with regards to the patients’ age. The age group of patients who used phytoestrogens was between 38 and 59 yr old (the mean age being 49.20 yr old), the age group of the patients on hormonotherapy were between 37–59 yr old (the mean age being 49.14 yr old), and the control group were of ages between 38–60 yr old (the mean age being 49.71 yr old). The majority of the patients included in the study were of over 50 yr of age. The presence of early menopause (at age under 40 yr old) was observed in over 11% in all 3 groups (11.29% in the group treated with phytoestrogens, 12.63% in the group treated by using hormonal therapy, and 11.32% in the control group).

Regardless of the age of the initial evaluation, there were no significant differences between the 3 groups considering the aspect of the severity of the somatic-vegetative symptoms (*P*=0.601 for age <40 yr, *P*=0.239 for age between 41–50 yr, *P*=0.772 for age >50 yr) (Table 1) (5).

**Table 1:** Prevalence of the severity of the somatic-vegetative symptoms on the initial evaluation, according to age

***Somatic-vegetative symtoms***	***Patients***					
	***< 40 yr***		***41–50 yr***		***> 50 yr***	
	***N***	***%***	***N***	***%***	***N***	***%***
Group on phytoestrogens therapy						
Light	1	7.14	7	13.46	20	34.48
Moderate	3	21.43	15	28.85	20	34.48
Severe	10	71.43	30	57.69	18	31.03
Total	14	100.00	52	100.00	58	100.00
Group on HRT						
Light	1	8.33	6	14.29	12	29.27
Moderate	3	25.00	12	28.57	15	36.59
Severe	8	66.67	24	57.14	14	34.15
Total	12	100.00	42	100.00	41	100.00
Control Group						
Light	1	8.33	4	8.70	17	35.42
Moderate	3	25.00	14	30.43	15	31.25
Severe	8	66.67	28	60.87	16	33.33
Total	12	100.00	46	100.00	48	100.00

Somatic-vegetative symptoms at ages under 40 are perceived as being more severe than in older age. At ages under 40, the severe somatic-vegetative symptoms are reported by 71.43% of the patients from the group treated with phytoestrogens, and 66.67% of the ones treated with HRT, and 66.67% of the control group; a significantly higher percentage compared to those over 50 yr of age (31.03%, 34.15%, 3.33%, respectively, *P*<0.001). The evolution of the somatic-vegetative symptoms is presented in Table 2.

**Table 2:** The evolution of the cumulated somatic-vegetative symptoms

***Somatic-vegetive symtoms***	***Group treated with Phytoestrogens***				***Group treated with HRT***				***Control Group***			
	***At 6 months***		***At 12 months***		***At 6 months***		***At 12 months***		***At 6 months***		***At 12 months***	
	***Patients***											
	***N***	***%***	***N***	***%***	***N***	***%***	***N***	***%***	***N***	***%***	***N***	***%***
Absent	44	35.48	54	43.55	31	32.63	38	40.00	25	23.58	39	36.79
Improved	57	45.97	50	40.32	44	46.32	39	41.05	36	33.96	27	25.47
No change	23	18.55	20	16.13	20	21.05	18	18.95	45	42.45	40	37.74

The disappearance (the absence) of the somatic-vegetative symptoms after 6 months was recorded at 35.48% of the patients from the group treated with phytoestrogen, a percentage slightly higher but not significantly higher in the group treated with HRT (32.63%) (*P*=0.543), but significantly higher than in the control group (23.58%) (*P*=0.005) The disappearance of the somatic-vegetative symptoms (score 0) was significantly higher in the group with HRT compared to the control group (*P*=0.0333).

At 12 months, the disappearance of the somatic-vegetative symptoms was recorded at 43.55% on the phytoestrogen therapy treated group, percentage slightly higher than in the group on hormonal therapy (40%) (*P*=0.469) and compared to the control group (36.79%) (*P*=0.161). There were no significant differences between the group on HRT and the control group on the disappearance of the somatic-vegetative symptoms (*P*=0.469)

The evolution of the somatic-vegetative symptoms was better in groups treated either with phytoestrogens or HRT (*P*<0.001) as opposed to the higher percentage of patients with stationary symptoms in the control group (i.e. 37.74% for control group, 16.13% for the group treated with phytoestrogens, respectively 18.95% for the group treated with HRT, *P*<0.001).

**Fig. 1:**
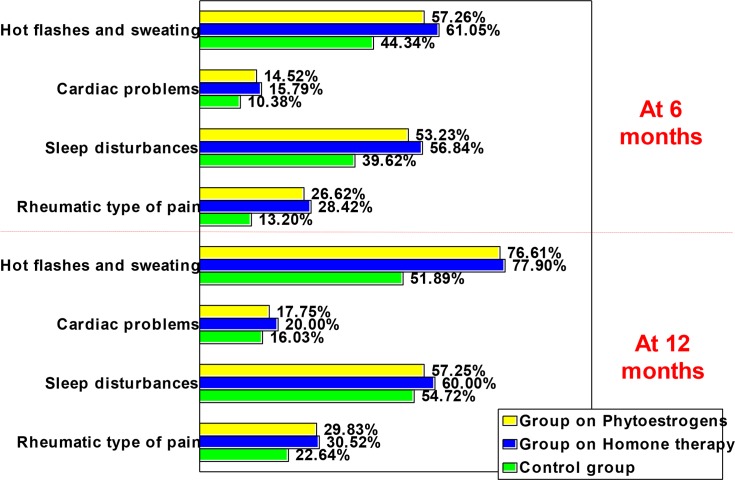
The favorable evolution (dissapearing + improvement) of the somatic-vegetative symtoms for each item

The evolution of the somatic-vegetative symptoms, on the evaluated items in MRS, at 6 months and at 12 months, is presented in Table 3. At 6 months evaluation, the evolution was favorable (disappearance of symptoms or improvement) especially in the hot flashes (57.26% for the group treated with phytoestrogens, 61.05% for the group treated with HRT, and 44.34% for the control group), followed by sleep disturbances (53.23% for the group treated with phytoestrogens, 56.84% for the group treated with HRT, and 39.62% for the control group). The same situation is encountered on the 12 months evaluation: favourable evolution seen especially in hot flashes (76.61% for the group treated with phytoestrogens, 77.90% for the group treated with HRT, and 51.89% for the control group), and regarding sleep disturbances (57.25% for the group treated with phytoestrogens, 60.00% for the group treated with HRT, and 54.72% for the control group) (Fig. 1).

**Table 3:** The evolution of the somatic-vegetative symptoms for each item

***Somatic-vegetive symtoms***	***Phytoestrogens Group***				***HRT Group***				***Control Group***			
	***At 6 months***		***At 12 months***		***At 6 months***		***At 12 months***		***At 6 months***		***At 12 months***	
	***Patients***											
	***N***	***%***	***N***	***%***	***N***	***%***	***N***	***%***	***N***	***%***	***N***	***%***
Hot flashes and sweating												
Absent	42	33.87	55	44.35	35	36.84	44	46.32	23	21.70	37	34.91
Improved	29	23.39	40	32.26	23	24.21	30	31.58	24	22.64	18	16.98
No change	53	42.74	29	23.39	37	38.95	21	22.11	58	54.72	48	45.28
Worse	0	0	0	0	0	0	0	0	1	0.94	3	2.83
Cardiac Problems												
Absent	12	9.68	15	12.10	11	11.58	14	14.74	6	5.66	13	12.26
Improved	6	4.84	7	5.65	4	4.21	5	5.26	5	4.72	4	3.77
No change	102	82.26	96	77.42	77	81.05	72	75.79	89	83.96	80	75.47
Worse	4	3.23	6	4.84	3	3.16	4	4.21	6	5.66	9	8.49
Sleep disturbances												
Absent	37	29.84	47	37.90	30	31.58	36	37.89	22	20.75	30	28.30
Improved	29	23.39	24	19.35	24	25.26	21	22.11	20	18.87	28	26.42
No change	57	45.97	52	41.94	40	42.11	37	38.95	63	59.43	46	43.40
Worse	1	0.81	1	0.81	1	1.05	1	1.05	1	0.94	2	1.89
Rheumatic type of pains												
Absent	18	14.52	24	19.35	16	16.84	20	21.05	7	6.60	17	16.04
Improved	15	12.10	13	10.48	11	11.58	9	9.47	7	6.60	7	6.60
No change	90	72.58	84	67.74	68	71.58	64	67.37	90	84.91	76	71.70
Worse	1	0.81	3	2.42	0	0	2	2.11	2	1.89	6	5.66

## Discussion

HRT significantly reduced the climacteric syndrome, even when administered in small doses (10). A Cochrane Group meta-analysis shows that systemic estroprogesterone HT significantly reduces the frequency and severity of hot flashes compared to placebo (13); the results of this study are consistent with literature data.

A systematic review and a meta-analysis of soy isoflavones vs. placebo in the treatment of vasomotor symptoms in postmenopausal women concluded that there was a strong tendency in favor of the soy-based products [14]. Hot flashes in menopausal women were decreased *P* to 50% with the consumption of 30 mg/day of soy isoflavones (or at least 15 mg Genistein) (15).

In this study, the statistic shows significant differences after six months, as well as at one year after initiation of HRT and the treatment with soy isoflavones, compared to the manifestations described by the patients before the therapy, with the improvement of all somatic-vegetative symptoms. The phytoestrogen and the hormone replacement therapy had a good effect on the attenuation of the symptoms (5).

Optimal results of the treatment for the two groups (treated with HRT and phytoestrogens) occur after 12 months of treatment. The treatment with hormones had slightly higher efficiency than with phytoestrogens. Similar results were obtained in other studies (14–16).

## Conclusion

All the somatic-vegetative symptoms assessed had a positive response using both therapies, with no major differences in efficacy between the treatment with phytoestrogens and the treatment with HRT. The 12 months treatment study is a favorable evidence for the use of soy-derived phytoestrogens for the treatment of somatic-vegetative symptoms at postmenopausal women.

## Ethical considerations

Ethical issues (Including plagiarism, informed consent, misconduct, data fabrication and/or falsification, double publication and/or submission, redundancy, etc.) have been completely observed by the authors.

## References

[1] Al-Eassa Abeer A Al-Fadel Abeer M Al-Ajmi Maryam A (2012). Knowledge and attitude of primary care doctors towards management of postmenopausal symptoms. Alexandria Med J, 48(2):167–73.

[2] Luoto R (2009). Hot flushes and quality of life during menopause. BMC Womens Health, 9:13. 19450250 10.1186/1472-6874-9-13 PMC2689180

[3] Polo-Kantola P Saaresranta T Polo O (2001). Aetiology and treatment of sleep disturbancies during perimenopause and postmenopause. CNS Drugs, 15(6):445–52. 11524023 10.2165/00023210-200115060-00003

[4] Wulf HU (2005). Psychosocial and socioeconomic burden of vasomotor symptoms in menopause: A comprehensive review. Health Qual Life Outcomes, 3:47. 16083502 10.1186/1477-7525-3-47 PMC1190205

[5] Ţiţ DM (2014). Comparative study on the effects of hormone replacement therapy (HRT) and phytoestrogens in the prevention of postmenopausal osteoporosis [PhD Thesis]. University of Oradea, Oradea.

[6] Palacios S (2008). Advances in hormone replacement therapy: making the menopause manageable. BMC Womens Health, 8:22. 19038018 10.1186/1472-6874-8-22 PMC2605606

[7] Bumbu A Paşca B Ţiţ D M Bungău S Bumbu G (2016). The effects of soy isoflavones and hormonal replacing therapy on the incidence and evolution of postmenopausal female urinary incontinence. Farmacia, 64(3): 419–22.

[8] Hsia J Simon JA Lin F (2000). Peripheral arterial disease in randomized trial of estrogen with progestin in women with coronary heart disease: the Heart and Estrogen/Progestin Replacement Study. Circulation, 102(18):2228–32. 11056097 10.1161/01.cir.102.18.2228

[9] Rossouw JE Anderson GL Prentice RL (2002). Risks and benefits of estrogen plus progestin in healthy postmenopausal women, principle results from the Women’s Health Initiative randomized controlled trial. JAMA, 288(3):321–33. 12117397 10.1001/jama.288.3.321

[10] Crandall C (2003). Low dose estrogen therapy for menopausal women: a review of efficacy and safety. J Womens Health (Larchmt), 12 (8):723–47. 14588124 10.1089/154099903322447701

[11] Stevenson JC Panay N Pexman-Fieth C (2013). Oral estradiol and dydrogesterone combination therapy in postmenopausal women: Review of efficacy and safety. Maturitas, 76(1):10–21. 23835005 10.1016/j.maturitas.2013.05.018

[12] Bungău S Ţiţ DM Fodor K Purza L (2015). The Treatment with Hormone Replacement Therapy and Phytoestrogens and the Evolution of Urogenital Symptoms in Postmenopausal Women. EC Pharm Sci, 1(3): 126–32.

[13] Grodstein F Manson JE Stampfer MJ (2006). Hormone therapy and coronary heart disease: the role of time since menopause and age at hormone initiation. J Womens Health (Larchmt), 15(1):35–44. 16417416 10.1089/jwh.2006.15.35

[14] Bolaños R Del Castillo A Francia J (2010). Soy isoflavones versus placebo in the treatment of climacteric vasomotor symptoms: systematic review and meta-analysis. Menopause, 17(3): 660–6. 20464785

[15] Kurzer MS (2008). Soy consumption for reduction of menopausal symptoms. Inflammopharmacology, 16(5): 227–29. 18815739 10.1007/s10787-008-8021-z

[16] Ţiţ DM Lazăr L Bungău S Pallag A Bei D (2013). The evaluation of the effectiveness of phytoestrogens in improving, reduction or suppression of the climacteric symptomatology. Studia Universitatis Vasile Goldiş Arad, Seria Ştiinţele Vieţii, 23(4): 585–89.

